# Celiac Disease and Glandular Autoimmunity

**DOI:** 10.3390/nu10070814

**Published:** 2018-06-25

**Authors:** George J. Kahaly, Lara Frommer, Detlef Schuppan

**Affiliations:** 1 Department of Medicine I, Johannes Gutenberg University (JGU) Medical Center, 55101 Mainz, Germany; Lara.Frommer@unimedizin-mainz.de; 2 Institute for Translational Immunology and Research Center for Immunotherapy (FZI), Johannes Gutenberg University (JGU) Medical Center, 55101 Mainz, Germany; Detlef.Schuppan@unimedizin-mainz.de; 3 Division of Gastroenterology, Beth Israel Deaconess Medical Center, Harvard Medical School, Boston, MA 02215, USA

**Keywords:** celiac disease, glandular autoimmunity, autoimmune thyroid disease, type 1 diabetes, polyglandular autoimmune syndrome

## Abstract

Celiac disease is a small intestinal inflammatory disease with autoimmune features that is triggered and maintained by the ingestion of the storage proteins (gluten) of wheat, barley, and rye. Prevalence of celiac disease is increased in patients with mono- and/or polyglandular autoimmunity and their relatives. We have reviewed the current and pertinent literature that addresses the close association between celiac disease and endocrine autoimmunity. The close relationship between celiac disease and glandular autoimmunity can be largely explained by sharing of a common genetic background. Further, between 10 and 30% of patients with celiac disease are thyroid and/or type 1 diabetes antibody positive, while around 5–7% of patients with autoimmune thyroid disease, type 1 diabetes, and/or polyglandular autoimmunity are IgA anti-tissue transglutaminase antibody positive. While a gluten free diet does not reverse glandular autoimmunity, its early institution may delay or even prevent its first manifestation. In conclusion, this brief review highlighting the close association between celiac disease and both monoglandular and polyglandular autoimmunity, aims to underline the need for prospective studies to establish whether an early diagnosis of celiac disease and a prompt gluten-free diet may positively impact the evolution and manifestation of glandular autoimmunity.

## 1. Celiac Disease

Celiac disease (CeD) is defined as a life-long intolerance to dietary gluten that results in small intestinal inflammation, villous atrophy, crypt hyperplasia, and often malabsorption. The ingestion of gluten containing cereal grains, mainly wheat, rye, and barley, drives this T cell driven auto-destructive process within the small intestinal mucosa which usually recovers when these cereals and gluten are rigorously withdrawn from the diet [1,2,3,4].

At least 50% of CeD is diagnosed in adulthood, and in the majority of adolescents and adults clinical features at diagnosis are subtle, with mild abdominal discomfort, fatigue, low bone mineralisation and hypocalcaemia, and only rarely manifest anemia, weight loss, infertility, or recurrent aphtous stomatitis. However, up to one third of adults suffer from one or more CeD-associated autoimmune diseases, prominently with autoimmune thyroid disease (AITD) and type 1 diabetes mellitus (T1D), but also rheumatoid diseases including systemic lupus erythematodes, Sjoegren’s syndrome, autoimmune liver diseases, and others [5]. Severe complications, like refractory CeD type 2, a premalignant condition, and overt enteropathy-associated T-cell lymphoma, occur in patients with longstanding undetected and untreated CeD, but remain rare [6,7]. Iron, zinc, vitamin D, vitamin B12, or folic acid deficiency, iron deficiency or overt anemia are the most common laboratory finding. Frequent episodes of hypoglycemia, a reduction of insulin requirements and brittle diabetes may indicate the presence of CeD in patients with T1D [8]. CeD is considered sufficiently prevalent and the benefits of diagnosis and treatment by gluten withdrawal are such that it is advocated to screen all patients with T1D (and autoimmune thyroid disease) for this disorder.

Both endoscopic-histological diagnosis and the presence of circulating IgA antibodies (Ab) to tissue transglutaminase (TG2) confirm the diagnosis. As shown in Table 1, anti-transglutaminase antibodies may be of IgG isotype in the presence, but also in the absence of a selective IgA deficiency. This suggests that the gluten-triggered autoantibody response shows mucosal IgA as its main component, while systemic IgG may represent a long-term reaction probably related to the occurrence of extra-intestinal manifestations. Consistently, it has been reported previously that the prevalence of CeD in T1D increases dramatically when the detection of both IgA and IgG autoantibodies is used in the screening. After a gluten-free diet the IgA-TG2-Ab disappear in most patients with CeD, usually with a half-life between 30 and 60 days. 

Genetic factors greatly determine susceptibility to CeD. All CeD patients carry HLA DR3/DQ2 (mainly DQA1*0501-DQB1*0201; 85–95%), or HLA DR4/DQ8 (DQA1*0301-DQB1*0302; 5–15%), or both haplotypes [1,2,3]. Exceptions are certain Native American populations that mainly carry DQ8 [9]. Since, e.g., the prevalence of HLA DQ2 in most populations is between 25 and 50%, only a minority with this necessary but insufficient genetic predisposition will ever develop CeD. This implies the involvement of additional, non-HLA linked genes, as well as environmental factors in CeD manifestation, as discussed below. 

The ubiquitous enzyme TG2, the CeD autoantigen, is central to the pathogenesis of CeD, since it can deamidate specific glutamine residues in certain gluten peptides that remain undigested and reach the subepithelial small intestinal lamina propria. This deamidation of the gluten peptides and their haptenization by binding to TG2 itself (autocatalysis) thereby increases their affinity to DQ2 or DQ8 on professional antigen presenting cells like macrophages, dendritic, and B cells, favoring the subsequent gluten specific destructive T cell response [1,10,11,12].

## 2. Monoglandular and Polyglandular Autoimmunity

Patients with CeD show a high prevalence of glandular autoimmune disorders [13,14,15,16]. CeD is associated with T1D, AITD i.e., Hashimoto’s thyroiditis (HT), Graves’ disease (GD), and the polyglandular autoimmune syndrome (PAS) [17].

### 2.1. Type 1 Diabetes

T1D is a T-cell mediated glandular autoimmune disease that develops in genetically susceptible individuals and results in destruction of the insulin-producing β cells. Of T1D patients, 15–30% have AITD and 3–12% present with CeD [18]. The close relationship between CeD and glandular autoimmunity can be widely explained by sharing of a common genetic background. However, some common pathogenic mechanisms have been further implicated, such as increased intestinal permeability resulting from zonulin upregulation and dysfunction of tight junctions in both CeD and T1D [19]. T1D is characterized by the infiltration of the pancreatic islets by lymphocytes and macrophages, the presence of autoantibodies to *islet cell antigens (ICA)*, *tyrosine phosphatase (IA2), glutamic acid decarboxylase-65 (GAD), insulin (IAA),* and *zinc transporter ZnT8 (Slc30A8)*, an increased prevalence of organ-specific autoimmune disorders in T1D, a preferential occurrence of T1D in persons carrying specific allelic combinations at immune response loci within the *HLA* gene complex. The disease can be transferred by spleen or bone marrow cells and animal models of T1D show a defect in immunoregulation contributing to the onset of disease [20].

### 2.2. Hashimoto’s Thyroiditis

HT is currently the most common autoimmune disease and frequently clusters with other autoimmune endocrinopathies. It is defined by the presence of thyro-peroxidase (TPO) or thyroglobulin (Tg) Ab and either normal or elevated serum thyroid stimulating hormone (TSH) concentrations. The majority of patients with HT are hypothyroid; however there is a subgroup of thyroid Ab-positive cases who are euthyroid. HT frequently occurs with T1D, with a prevalence of 13–20% of subclinical hypothyroidism in T1D patients compared with 3–6% in a non-diabetic population. Overt hypothyroidism is present in 4–18% of subjects with T1D. TPO-Ab are present in 15–30% of adults and in 5–22% of children with T1D, compared with 2–10% and 1–4%, respectively, in healthy controls. Up to 50% of TPO-Ab positive T1D patients progress to overt AITD. As many as 30% of patients with T1D develop AITD. Age, duration of T1D, and female preponderance impact the link between T1D and AITD [21,22]. In a prospective controlled study, celiac patients had an increased risk of thyroid autoimmune disorders when compared to non-celiac controls on normal gluten-containing diet [23]. However, in this Scandinavian trial, a gluten-free diet seemed not to prevent the progression of autoimmune process during a follow-up of one year.

### 2.3. Graves’ Disease

GD is meanwhile less prevalent than HT; it affects approximately 1–1.5% of the general population worldwide and is the underlying cause of 50–80% of cases of thyrotoxicosis. GD and HT share many immunological features, including high serum concentrations of Ab against Tg and TPO. GD is caused by TSH receptor stimulating Ab [24,25,26] that bind to and activate the TSH receptor on thyroid cells. These Ab not only cause hypersecretion of thyroid hormone, but also promote hypertrophy and hyperplasia of thyroid follicles, resulting in an enhanced vascularization of the gland and in a diffuse goiter. Women are five to ten times more at risk of developing GD than men, due to the relevant involvement of sex hormones. Also, stress and negative life events are regarded as risk factors for GD and may trigger the disease. Subclinical endogeneous hyperthyroidism can be diagnosed in 6–10% of T1D patients, compared with 0.1–2% in the non-diabetic population. The incidence of overt autoimmune hyperthyroidism in persons with a suppressed serum TSH is calculated at 2–4% per year [27].

### 2.4. Polyglandular Autoimmune Syndrome

The association of two or more glandular autoimmune diseases is designated as PAS. Especially in adults, the presence of one autoimmune endocrine disorder increases the risk of developing other autoimmune diseases. CeD is a strong predictor not only of glandular but also polyglandular autoimmunity. PAS shows a great heterogeneity of syndromes and usually manifests sequentially, with a variable time interval between the occurrence of the first and second glandular autoimmune disease component. It also clusters with several non-endocrine autoimmune diseases [28]. PAS is divided into two major subtypes, which are distinguished according to age of presentation, characteristic patterns of disease combinations and different modes of inheritance. Juvenile PAS I, also known as autoimmune polyendocrinopathy-candidiasis-ectodermal dystrophy, usually manifests in infancy or childhood at age three to five years, or in early adolescence, It is characterized by a persistent fungal infection (chronic mucocutaneous candidiasis), the presence of acquired hypoparathyroidism and adrenal failure. In most patients mucocutaneous candidiasis precedes the other immune disorders, usually followed by hypoparathyroidism. The female-to-male ratio varies from 0.8:1 to 2.4:1. The highest prevalence of PAS I has been found in populations characterized by a high degree of consanguinity or descendants of small founder populations, particularly in Iranian Jews and Fins. PAS I is a monogenic disease with autosomal recessive inheritance caused by mutations in the autoimmune regulatory gene (AIRE) on chromosome 21. Adult PAS occurs mainly in the third or fourth decade. Adult PAS subtype II encompasses adrenal failure or Addison’s disease (AD) with other autoimmune endocrine disorders, i.e., AITD and/or T1D. AD may precede other endocrinopathies. In contrast to the juvenile type, family members of adult PAS II patients are often affected. PAS II is believed to be polygenic, characterized by autosomal dominant inheritance [29,30,31,32,33,34].

The adult PAS type III is the most frequent PAS type and is characterized by AITD and T1D and the absence of AD. In contrast, the poorly defined PAS type IV is very heterogeneous involving a large variety of glandular autoimmune diseases that are not considered within adult PAS types 2 and 3. Being less well defined, it is often incorrectly described as a combination of monoglandular autoimmune disease with one non-glandular autoimmune disease. In fact, PAS type IV includes various combinations of autoimmune hypopituitarism, hypergonadotropic hypogonadism, or hypoparathyroidism with T1D or an AITD. The adult form of PAS has a prevalence of 1:20,000, and is far more prevalent than the juvenile type, with an annual incidence of 1–2:100,000. The gender ratio of adult PAS types II–IV shows a female predominance of 75% [35,36]. The manifestation peaks in the fourth and fifth decade depending on the combination of the various autoimmune endocrinopathies [37].

Even if the time between the manifestations of CeD and other autoimmune endocrinopathies, as well as the time until a PAS can be diagnosed, is highly variable, many patients with one autoimmune disease already have Ab against other, often endocrine, tissues (Table 2). The reason is the tendency of autoimmune diseases to associate with one another, especially when they share a genetic basis, of the metachronous manifestations of the component diseases, and of the often subclinical initial course. Mainly first but also second degree family members of patients are often at risk for developing related autoimmunities and are already Ab positive.

## 3. The Role of a Gluten Free Diet in Preventing Celiac Disease and Glandular Autoimmunity

A large prospective study demonstrated that in infants at genetic risk for CeD and T1D (i.e., from families with at least one affected parent and the *DR3/DQ2* and/or *DR4/DQ8* risk genes) a careful early introduction of 100 mg gluten per day in the diet from month 5–6 did not prevent celiac autoimmunity compared to placebo [45]. Moreover, the introduction of gluten at 12 instead of 6 months of age only delayed the onset of CeD, with similar prevalences at age 5 years [46]. However, a retrospective study indicated that patients on a long-term gluten free diet developed 50% fewer autoimmune diseases in up to 15 years of follow up [47]. The (retrospective) studies that examined the effect of a gluten free versus gluten containing diet on the development and severity of T1D and AITD remain controversial (Table 3 and Table 4). Interestingly, T1D appears to precede the development of CeD, as determined by IgA-TG2 Ab positivity, which would assign a less important role to the gluten free diet in the prevention of glandular autoimmunity [19].

Several mainly retrospective and correlative studies, often based on registries, have tried to address the question, how far an early diagnosis of CeD and/or a gluten free diet may protect from AITD or T1D. As shown in tables 3 and 4, in these studies early diagnosis of CeD did not appear to protect from the development of T1D [42,48,49,50,51,52,53]; whereas some studies suggested such protection in AITD [42,48,54,55,56,57,58,59]. In comparison, a gluten free diet (GFD) may positively impact the occurrence of T1D rather than of AITD. These somewhat conflicting data need validation in large, prospective studies with well-defined diagnosis and markers of CeD, T1D, and AITD. Such studies are currently performed in children with an increased risk for the three diseases (being offspring of affected parents and carrying the *DQ2* and/or *DQ8* genes. However, a beneficial effect of a gluten free diet may be expected, since in general, in children as well as in adults, intestinal inflammation and the associated dysbiosis, with or without underlying CeD, are known to promote extra intestinal autoimmune diseases [60,61,62]. Therefore, any measure that would dampen gut inflammation in CeD patients will likely positively impact the evolution and perhaps the manifestation of glandular autoimmunity.

## Figures and Tables

**Table 1:** Autoimmune disease specific antibodies.

Disease	Autoantibodies
Celiac disease	Tissue transglutaminase IgA (IgG)
Type 1 diabetes	Glutamate decarboxylase (GAD) Tyrosine phosphatase (IA2) Islet cell (IC) Insulin Zinc transporter (ZnT8)
Autoimmune thyroid disease	Thyro-peroxidase (TPO) Thyroglobulin (Tg) TSH receptor

**Table 2:** Prevalence of autoimmune thyroid disease auto-antibodies in patients with celiac disease.

CeD Patients Studied (*N*)	Prevalence of Thyroid Autoantibodies, *N*	Prevalence of Thyroid Autoantibodies in %	Reference
107	Tg Ab, *N* = 12	11.2	[38]
	TMA, *N* = 16	15	
70	TMA, *N* = 15	21	[39]
47	TPO, *N* = 14	29.7	[40]
34	TMA, *N* = 5	14.7	[41]
	Tg Ab, *N* = 11	32.4	
	TPO, *N* = 4	11.8	
90	TPO, *N* = 13	14.4	[42]
36	TPO, *N* = 11	30.5	[43]

Modified according to reference [44]. CeD: celiac disease, Tg-Ab: thyroglobulin antibodies; TMA: thyroid microsomal antibodies; TPO-Ab: thyro-peroxidase antibodies.

**Table 3:** Gluten exposure and occurrence of type 1 diabetes.

Patients (*N*)	Duration of Follow-up (Years)	Early CeD Dx Protective?	GFD Protective?	Reference
90	2	NE	Yes	[42]
44	1.6	No	Yes	[48]
383	7.6	No	NE	[49]
1183	0.9	NE	Yes	[50]
19796	NE	No	NE	[51]
4322	NE	No	NE	[52]
150	3	NE	Yes	[53]

**Table 4:** Gluten exposure and occurrence of autoimmune thyroid disease (AITD).

Patients (*N*)	Duration of Follow-up (Years)	Early CeD Dx Protective?	GFD Protective?	Reference
909	>0.5	Yes	NE	[42]
44	1.6	No	Yes	[48]
66	1–5	Yes	Yes	[54]
343	0.25–16	NE	No	[55]
324	8	Yes	No	[56]
135	8.9	No	No	[57]
545	2	No	No	[58]
335	9	No	No	[59]

Dx: diagnosis; GFD: gluten free diet; NE: not evaluated.
